# Prevalence of Reversible and Potentially Reversible Cognitive Frailty in Older Adults: A Meta-Analysis

**DOI:** 10.1093/geroni/igaf122.2176

**Published:** 2025-12-31

**Authors:** Jinwei Bian, Zi Chen, Ying Gao, Fung Yee Lau, Daniel Yee Tak Fong, Edmond Pui Hang Choi, Pui Hing Chau

**Affiliations:** The University of Hong Kong, Hong Kong, China (People’s Republic); The University of Hong Kong, Hong Kong, China (People’s Republic); The University of Hong Kong, Hong Kong, China (People’s Republic); The University of Hong Kong, Hong Kong, China (People’s Republic); The University of Hong Kong, Hong Kong, China (People’s Republic); The University of Hong Kong, Hong Kong, China (People’s Republic); The University of Hong Kong, Hong Kong, China (People’s Republic)

## Abstract

**Background:**

Potentially reversible cognitive frailty (PRCF) and reversible cognitive frailty (RCF), characterized by concurrent declines in physical and cognitive function, may be modifiable or responsive to interventions. Understanding their epidemiology can inform research and shape public health strategies. However, reviews on their prevalence remain scarce. This study aimed to estimate the pooled prevalence of PRCF and RCF among older adults without dementia.

**Methods:**

A search of 6 literature databases was conducted from inception to August 15, 2024. Cohort and cross-sectional studies reporting PRCF or RCF prevalence in older adults aged 60+ without dementia were included. Random-effects meta-analyses with logit-transformed prevalence were used, alongside subgroup analyses and meta-regression to examine heterogeneity. Risk of bias was assessed using the Joanna Briggs Institute Critical Appraisal Checklist.

**Results:**

Of the 13,100 articles identified, 51 studies from 14 countries were eligible, with 50 studies on PRCF and 10 studies on RCF. The pooled prevalence of PRCF in communities, hospitals and nursing homes was 17% (95% CI: 13%-21%), 32% (95% CI: 23%-42%), and 32% (95% CI: 1%-99%), respectively, while the pooled RCF prevalence was 21% (95% CI: 15%-29%), 48% (95% CI: 45%-51%), and 15% (95% CI: 12%-19%), respectively. Studies with suboptimal sample sizes reported higher PRCF prevalence compared to those with optimal sample sizes.

**Conclusion:**

The prevalence of PRCF and RCF among older adults is substantial and varies across settings, highlighting the need for early detection and intervention. Further research in underrepresented regions, along with prior sample size calculations and appropriate assessment tool selection, is essential.

